# Effect of Filling Rate on Underwater Wet Welding Process and Weld Appearance

**DOI:** 10.3390/ma13051061

**Published:** 2020-02-27

**Authors:** Xin Zhang, Ning Guo, Changsheng Xu, Haoran Kan, Yanbo Tan, Hao Chen

**Affiliations:** 1 State Key Laboratory of Advanced Welding and Joining, Harbin Institute of Technology, Harbin 150000, China; zxin299@126.com (X.Z.); xchshah@163.com (C.X.); 18678673126@163.com (H.K.); b17863066695@163.com (Y.T.); chh523@126.com (H.C.); 2 Shandong Provincial Key Laboratory of Special Welding Technology, Harbin Institute of Technology at Weihai, Weihai 264209, China; 3 Shandong Institute of Shipbuilding Technology, Weihai 264209, China

**Keywords:** underwater wet welding, welding process, filling rate, droplet transfer mode

## Abstract

Real-time electric signal, matter transfer mode and welding pool behavior were obtained to investigate the effect of wires’ filling rate on arc stability and joints’ appearance during underwater wet flux-cored arc welding (FCAW). The electric signal results showed that arc stability first decreased and then increased rapidly because the raise of filling rate affected the number of charged particles and the electrical conductivity of welding arc atmosphere. Two typical transfer modes, globular repelled transfer mode and surface tension transfer mode, were observed in this study. The ratio of surface tension transition could be increased by adding wires’ filling rate. Meanwhile, the geometry of molten pool was changed and the distance between droplets to welding pool reduced as the filling rate increased. The fusion line became more regular and the radius of curvature increased under the effect of bubbles in the molten pool. As the filling rate improving, more slags on the welds surface were acquired and the welds were much flatter and smoother.

## 1. Introduction

Underwater welding is a rapidly developing key marine technology used in the repair and maintenance of ocean platforms, marine vessels and submarine pipeline [1,2]. There are three methods of underwater welding specific to welding environment: underwater wet welding (UWW), underwater local-dry welding and underwater dry welding [3]. As the most widespread underwater welding technology, UWW technology has developed comprehensively since ever invented by virtue of its maintainability, operability and consumability.

In the past century, the most common filling materials in UWW technology was covered from electrodes to flux-cored wires [4]. By adjusting the component and the relative content of electrode coating and core metal, high quality welded joints could be obtained in various commonly used structural steels and stainless steels [5]. However, there were several technical defects that restricted its development. The welding process could not achieve automation because the electrode was required to be replaced artificially and frequently. Project lifecycle was extended by the low efficiency and the welder was placed in a danger environment with high water pressure. The quality of the welding joint was greatly affected by the welder’s ability and lead to associated uncertainty. Therefore, a novel method, flux cored arc welding (FCAW) has become a new focus of research in recent decades.

The work efficiency and welded joints’ performance has drastically improved with underwater wet FCAW. The joints’ properties and appearance can be controlled by adjusting the powder composition and relative content of the compound and metal particles in the flux cored wires. CaF_2_ plays a significant role in dehydrogenation and appearance. Zhang et al. investigated the influence of CaF_2_ content on the welding performance and properties in UWW. The addition of CaF_2_ from 0% to 65% significantly improved the weld formation and the depth-width ratio descended from 33.5% to 15.7% [6]. Guo et al. believed that the appropriate content of Ni could improve the tensile strength of E40 steel joint, but excess Ni could cause the decrease of impact toughness [7]. Li et al. studied the effect of alumino-thermic addition on underwater wet welding process stability [8]. The thermite addition would improve the stability of the wet welding process while there was a lot of numerous tiny spatters on the surface of weld at larger content of thermite. Liu et al. investigated the effects of Mo, Ti and B on microstructure and mechanical properties of UWW joints indicating that although an excessive content of Mo, Ti and B in the deposited metal would induce an improvement of the weld strength, it would also lead to a sharp deterioration of the weld plasticity and toughness [9].

A more suitable welding process was more conducive to obtain high quality welded joints. Guo et al. observed four fundamental transfer modes in underwater FCAW: the globular repelled transfer mode, the surface tension transfer mode, the explosive short-circuit transfer mode and the “submerged arc transfer mode” pointing out that the previous two were the main transfer modes [10]. Jia et al. proposed the impeding effect of arc bubbles on droplet transfer process [11]. Chen et al. studied the behaviors and characteristics of arc bubble by an in-situ X-ray method. A three-stage model of the effect of bubble behaviors on droplet transfer was presented [12].

The content of powder has an enormous influence on the joints’ appearance, properties and welding process. Holding a uniform filling rate of the wires was a necessary condition. However, there were few literatures that indicated the influence of the powder filling rate on welding process and weld joints in underwater FCAW. Most of the flux-cored tubular wires used in underwater welding was manufactured in four steps: pressing groove, filling powder, squeezing into circular and reducing the diameter. Filling rate, represented by η, means the mass fraction of powder added in the groove in the filling powder step. It could be defined as the weight of the powder divided by the total weight of the powder and the coating. It could be derived as equation 1:

η = m1/(m1 + m2)(1)

where, m1 is the weight of powder per unit length, m2 is the weight of metal strap outside per unit length.

Wires with larger filling rate means more powder has melted and transferred into the molten pool during underwater FCAW. Thus, the welding beam will have more alloying elements and a thicker coating slag. On the other hand, the diameter of wires has typically been constant. A larger filling rate means a thinner metal strap outside as a contrast. In UWW process, the current was mainly passed by the outer metal. A thinner outer mater would reduce the effective conductive area for electronic and increase the electrical resistance. The heat quantities of elongation would change.

As stated above, filling rate cannot be ignored in relation to effect of powder components. In this study, a mature flux cored wire powder formula was selected and four welding wires with different filling rates were designed to evaluate the effect of filling rate addition on underwater wet welding process stability and weld appearance.

## 2. Materials and Experiments

**Figure 1:**
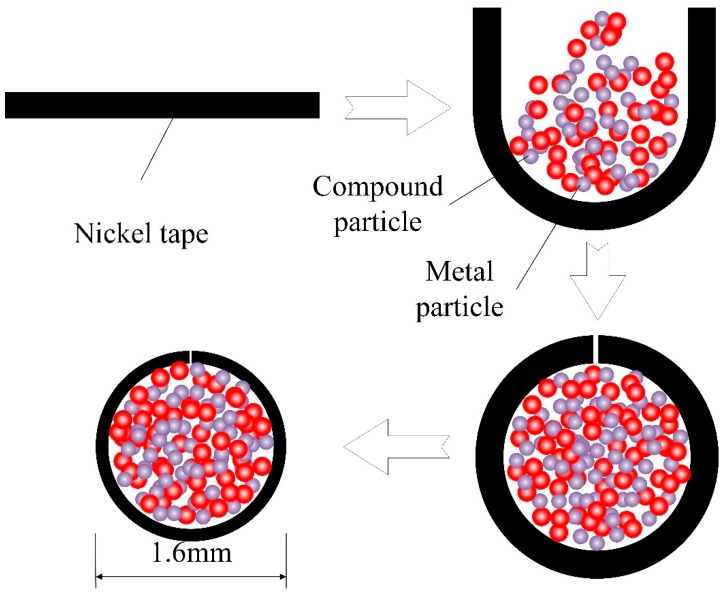
Flux-cored wires formation process.

A special self-protecting flux cored wire for underwater wet welding was employed in this experiment. The dimension of the pure nickel strap (99.99%) outside is 8 mm × 0.3 mm. The mixed powder inside is composed of slag-forming constituents, gas-forming constituents, arc stabilizer and thermite, which will produce an Al_2_O_3_ (about 20 Wt.%) − CaF_2_ (about 70 Wt.%) slag system. The main components of powder are shown in Table 1. In order to accurately analyze the effect of filling rate, the electrode wire with 1.6mm in diameter was filled with filling rate of 15, 20, 24 and 28% respectively. The formation process of wires included four steps: U-groove pressing formation, powder filling, rolling sealing and diameter reduction, as shown in Figure 1. The base metal used in this investigation was full-austenite 304 L stainless steel with a dimension of 200 mm × 150 mm × 10 mm.

A SAF-FRODIGI@WAVE500 welding source was used and the automatic welding process was operated underwater in 0.5m depth. Direct current electrode positive (DCEP) was used and welding parameters are exhibited in Table 2.

**Figure 2:**
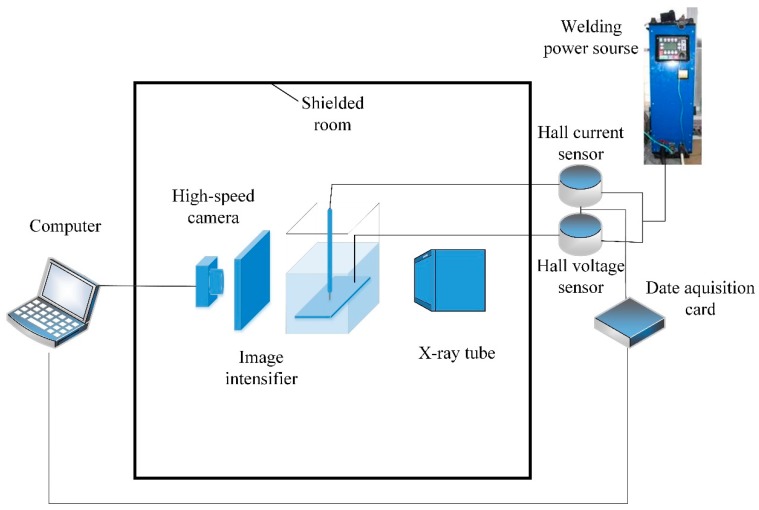
Experimental equipment schematic.

To precisely observe the droplet transfer behavior, an X-ray in-situ imaging system was utilized to capture the droplet transfer process image as shown in Figure 2. It consisted of a micro-focused X-ray tube, an image intensifier and a high-speed camera. The high-speed camera has a frame rate up to 5000 frames per second. The whole system was wrapped in a lead chamber to isolate X-rays. The welding current and arc voltage were recorded by a high-speed data acquisition system with a 10 kHz sampling rate in order to directly and timely reflect the welding stability.

## 3. Results and Discussion

In underwater wet FCAW, larger filling rate of wires related to thinner outer metals and more powder particle inside. Wires with different filling rates could cause changes in the electrical characteristic of arc, the droplet transfer mode, the welding stability and even the welded joints’ appearance.

### 3.1. The Variation of Electrical Signal Indexes

**Figure 3:**
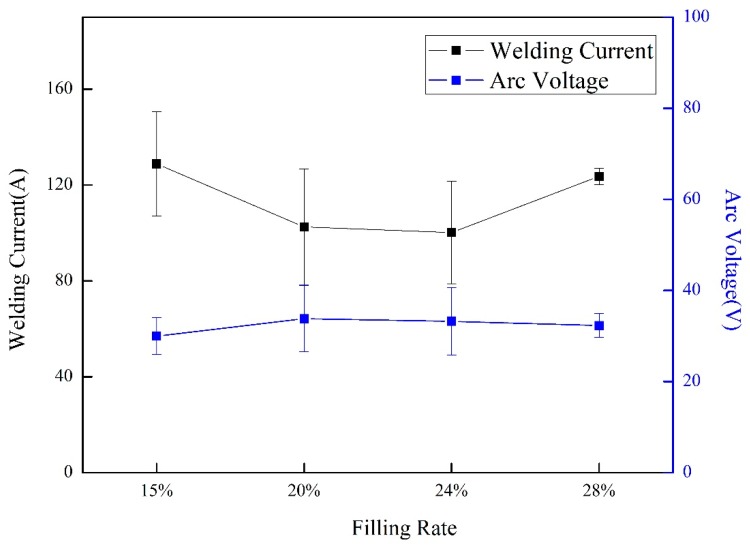
Effect of filling rate on average welding current.

**Figure 4:**
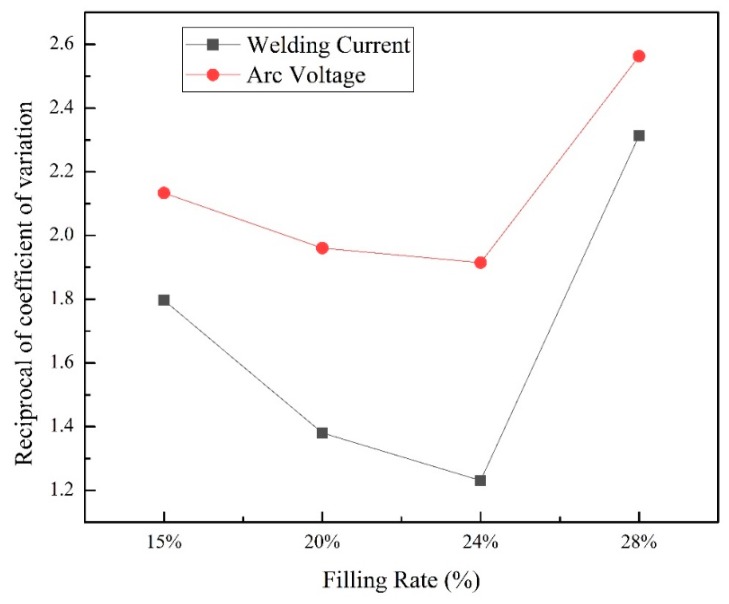
Reciprocal of variation coefficient at different filling rate.

Figure 3 shows the change of average welding current and arc voltage as filling rate growing from 15% to 28%. The average welding current of wires with different filling rate decreased from 129A to 97A as the filling rate increased from 15% to 24% and then grown to 127A at 28%. The error bar showed an opposite tend. In welding process, too much short circuits or arc extinctions were the fundamental cause of welding arc stability decline. When a short circuit occurred, smaller arc voltage and larger current occurred. When an arc extinction occurred, a small welding current and a large arc voltage occurred. In this study, the standard deviation was calculated based on the measured welding current and arc voltage. The variation coefficient of arc voltage (welding current) was the ratio of the standard deviation and the mean value of the measured arc voltage (welding current). Its reciprocal could more precisely reflect arc stability by the reciprocal of variation coefficient of arc voltage and welding current. A larger reciprocal of variation coefficient indicated a smaller standard deviation of arc voltage (welding current). This signified that there were less moments in the welding process that short circuit or arc extinction occurred. As shown in Figure 4, the reciprocal of current and voltage variation coefficient decreased as the filling rate adding from 15% to 24% and then sharply increased. It is indicated that the arc stability was deteriorated with more powder in wires. However, it became more stable as the filling rate increased.

**Figure 5:**
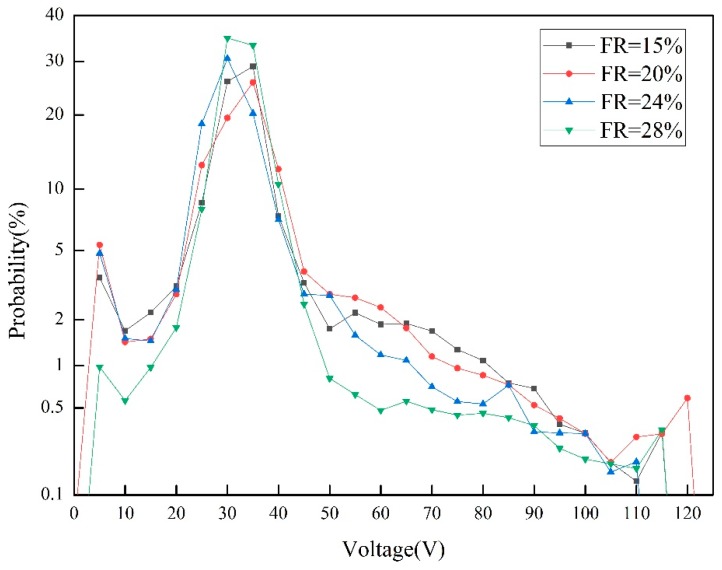
The effect of filling rate on probability of voltage distribution.

In this study, the probability of the arc voltage distribution was characterized as Figure 5. As illustrated by Chen [13], there were three regions in the arc voltage distribution probability diagram: the short-circuit region in left; the arc extinction region in right; the large hump in the center which represented the stable combustion of welding arc. An intense and narrow large hump were indicated a stable welding arc, and vice versa. When the filling rate was 28%, the curve was significantly lower than others in the short-circuit region and arc extinction region while the middle hump was also more concentrated and higher, which indicated a more stable welding arc.

### 3.2. Metal Transfer Process

**Figure 6:**
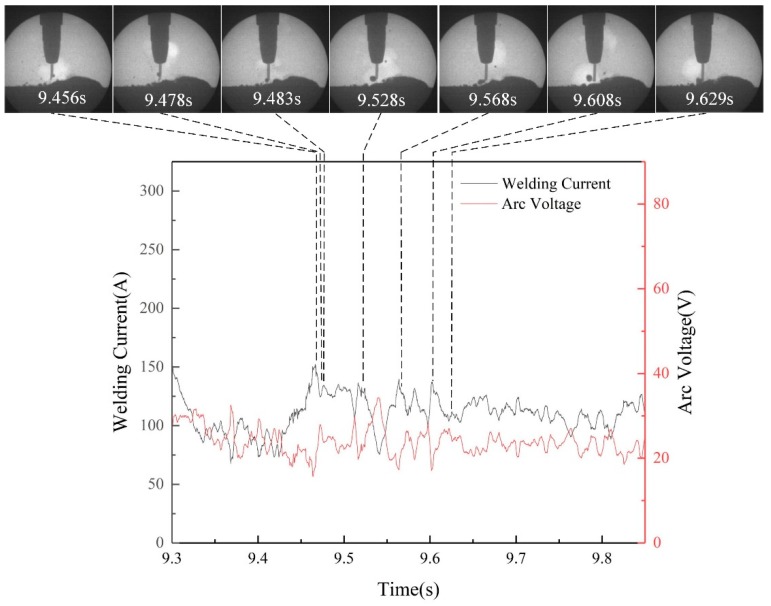
Typical globular repelled transfer mode and electric signal (28% filling rate).

The droplet transfer mode was closely related to the stability of welding process [14]. In this study, two transfer mode were observed in every filling rate by X-ray in-situ imaging system in the welding process. Taking the droplet transfer process with the filling rate of 28%, as an example, the typical globular repelled transfer process and the electrical signal were depicted in detail, as shown in Figure 6. At 9.456 s, the wire melted and a small droplet formed in the tip. As the wire continued to melt, the droplet continued to grow and sway up and down at the tip of the wire, causing the arc voltage to constantly change. At 9.568 s, the droplet left the tip of wire and entered into arc bubble. At 9.629 s, the droplet drip into the welding pool. The electrical signal photograph demonstrated that during the globular repelled transfer process, the welding current and arc welding were fluctuated in a tiny range.

**Figure 7:**
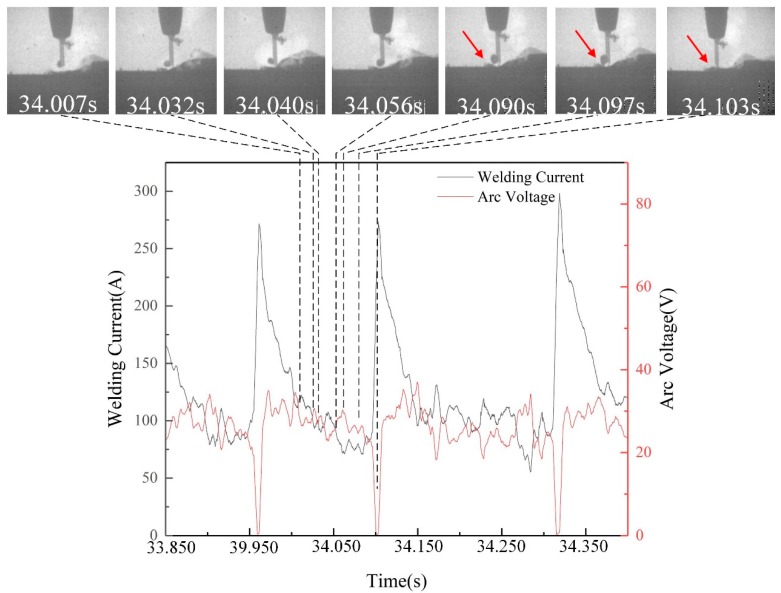
Typical surface tension transfer mode and electric signal (28% filling rate).

Figure 7 presented the surface tension transfer process and electrical signal when the filling rate was 28%. The surface tension transfer process could be divided into two steps: repulsion step and transfer step. From 34.007 s to 34.090 s, a repulsion step occurs in surface tension transfer process under the effect of the repulsive force. However, the droplet did not leave the end of the wire until it had contacted with the molten pool at 34.097 s and then entered the pool under the action of surface tension at 34.103 s. The electrical signal was the same as the repulsion transfer mode in the repulsion step. As long as the droplet contacted the molten pool surface, the welding current sharply increased to 275 A and the arc voltage harshly decreased to 0 V, like an arc extinction process.

**Figure 8:**
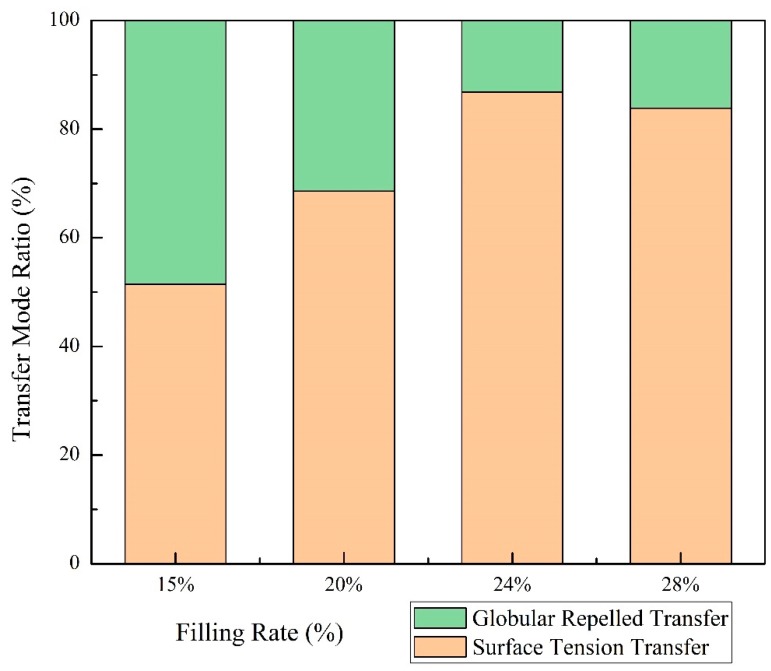
The effect of filling rate on the relative proportion of metal transfer mode.

**Figure 9:**
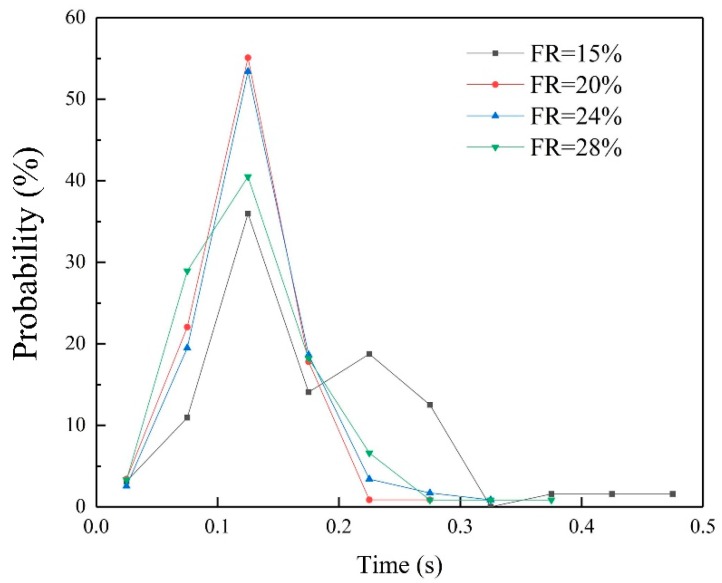
The effect of filling rate on probability of droplet growth time.

Figure 8 shows the effect of wires’ filling rate on the metal transfer mode. The percentage of surface tension transfer mode were always above 50% in this experiment, which were higher than globular repelled transfer mode. About 51.43% of the droplet transfer were processed in surface tension transfer mode, while it rose to 86.84% as the filling rate growing to 24%. Then the percentage of surface tension transfer mode dropped slightly to 83.87% when the filling rate was 28%. It could be considered that as long as the filling rate increases to a certain value (in this study, 24%), the proportion of different droplet transition modes tends to be stable and only fluctuates within a certain small range. Figure 9 shows the effect of filling rate on probability of droplet growth time. As the filling rate was 15%, a considerable number of droplets with a formation time more than 0.2 s was generated. When the droplet filling rate was 20%, the number of droplets whose growing time below 0.2 s increased. However, continuing to increase filling rate, the droplets’ growth time did not decrease significantly. The reason would be given in Section 3.4.

**Figure 10:**
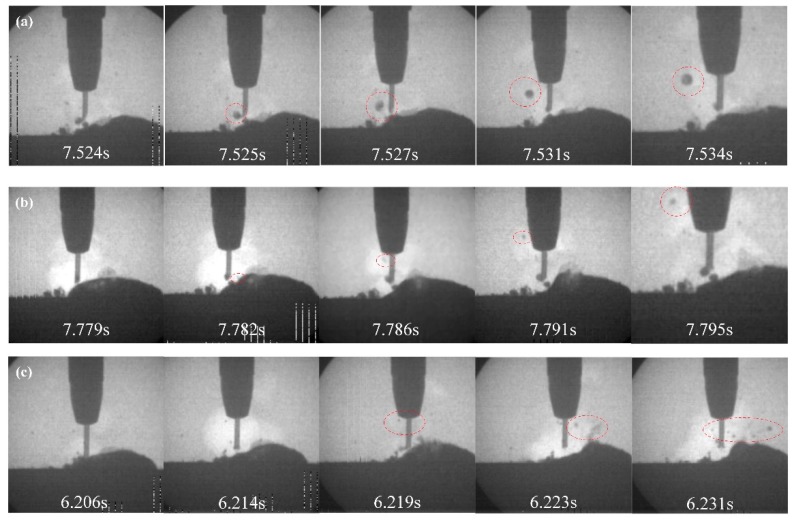
Spatters during metal transfer process at 15% filling rate. (**a**) Droplet repelled spatter. (**b**) Weld pool oscillation spatter. (**c**) Explosive spatter.

Additionally, three spatter types were observed in every welding process as shown in Figure 10. According to Fu et al. [15], the three kinds of spatter were droplet repelled spatter, weld pool oscillation spatter and explosive spatter. The droplet repelled spatter has a significant influence on the welding appearance because this kind of spatters with large diameter would fall on both sides of the weld metal and difficult to remove. The explosive spatter also has an obviously effect on weld appearance by causing the pool to vibrate violently.

### 3.3. Weld Appearances

**Figure 11:**
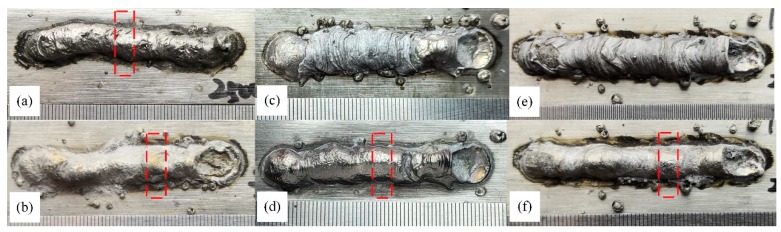
The effect of filling rate on weld appearance with different filling rate wires. (**a**) 15%; (**b**) 20%; (**c**) 24% (with slag); (**d**) 24% (without slag); (**e**) 28% (with slag); (**f**) 28% (without slag).

Figure 11 shows the appearance of welded joints acquired by wires with different filling rate. When the filling rate were 15% and 20%, on the weld surface was covered almost no slag as presented by Figure 11a,b. As the filling rate growing, there were more and more slags were formed as given in Figure 11b,c,e. At the same time, the quantity of spatters increased. The welded beams were more and more straight while the surface more glabrous and smoother after disengaging slags, as displayed in Figure 11a–d,f. According to these figures, the weld appearance was substantially improved with increasing the filling rate. It could be attributed to the protection of the more and more slags. The existence of molten slags reduced the fluctuation amplitude of molten pool surface. When the bubbles in the molten pool broken, it could also effectively inhibit the metal to fly out of the molten pool. However, as the stability of the arc first decreased and then increased, the arc force of the droplet was not stable, and the droplet repelled spatter increased.

**Figure 12:**
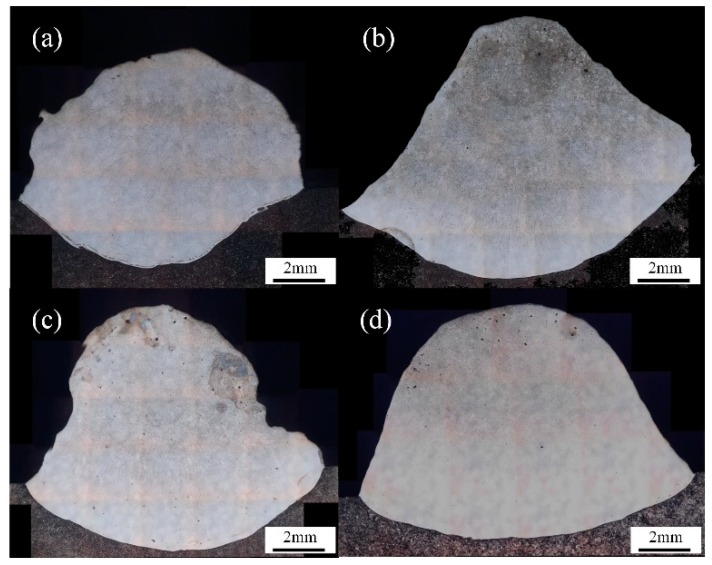
The cross sections of welding joints with different filling rate. (**a**) 15% filling rate; (**b**) 20% filling rate; (**c**) 24% filling rate; (**d**) 28% filling rate.

The metallographic sample was taken according to the position shown by the red dotted line in Figure 11. After polishing and corrosion, the cross sections shown in Figure 12 were obtained. Figure 12 shows the cross sections of welding joints produced at different filling rate after etching. Obviously, the outline became much more regular. The number of defects, such as blowholes and slag inclusion, also decreased as the filling rate increased from 15% to 28%. The fusion line in Figure 12a was irregular arc while the others were regular with a larger and larger radius, as shown in Figure 12b–d.

**Figure 13:**
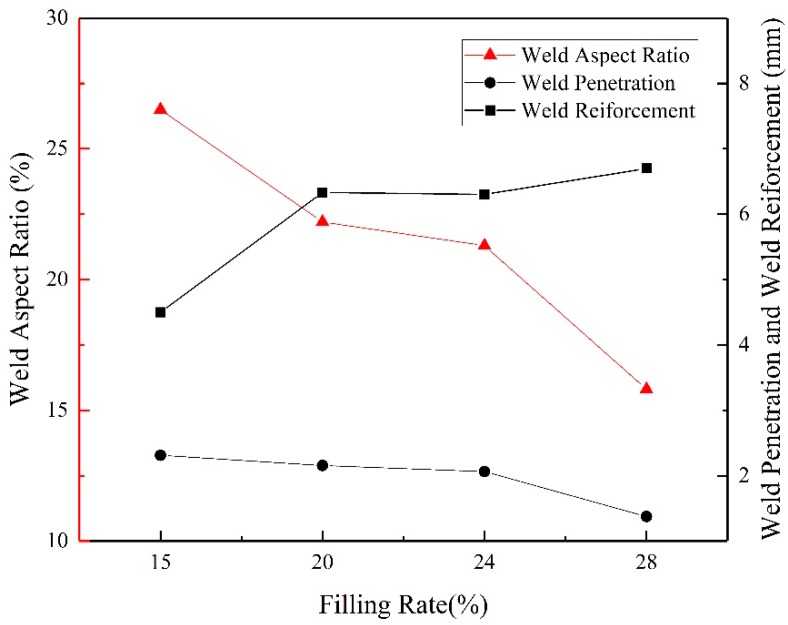
The effect of filling rate on weld aspect ratio, weld penetration and reinforcement.

Aspect ratio was an important parameter of weld formation. The ratio of weld depth to weld width could reflect the characteristics of weld formation to a certain extent. Figure 13 illustrated the weld penetration depth, the aspect ratio and the reinforcement by calculation. The weld aspect ratio varied from 26.5% to 15.8% as filling rate increased from 15% to 28%. Since the weld width has just little fluctuation, the reduction of weld dilution rate was mainly caused by weld penetration depth. The weld penetration depth decreased as filling rate increased which was the result of a combination of factors.

### 3.4. Influence Mechanism Analysis of Thermite

Three aspects, electrical signal indexes, metal transfer process and weld appearances, were used to evaluate the effect of wire’s filling rate on UWW process. The mechanism analysis would be discussed in the following three aspects including arc electrical properties, the forces acting on the droplets in the molten electrode tip and molten pool behavior.

Firstly, the effect of filling rate on the welding arc stability was mainly the change of electric conductivity of arc plasma atmosphere. As shown in Table 1, CaF_2_, as an important dehydrogenation composition [16], accounted for 60% of the mass of the powder. Under the high temperature of arc, CaF_2_ molecular would decompose into F atom and Ca atom (Formula 2). The F atom was very easily to absorb free electrons in the arc atmosphere and formed F^−^ (Formula 3). The F^-^ could combine with H^+^ and the conductivity of the arc atmosphere decreased (Formula 4) [16].

CaF_2_ → Ca + 2F (2)

F + e^−^ → F^−^ (3)

F^−^ + H^+^ → HF (4)

When the filling rate increased from 15% to 24%, the consumption of charged particles continued to strengthen. The number of e^-^ in the welding arc atmosphere continued to decrease. The conductivity of the arc atmosphere decreased, which caused the decrease of arc stability. However, continuing to increase the filling rate brought in enough CaF_2_, which made the reaction in Formula 3 reached saturation. Free electrons were no longer scarce, and excess ions, such as F^−^ and H^+^, could enhance electrical conductivity in arc atmosphere. Finally, the arc stability was improved.

**Figure 14:**
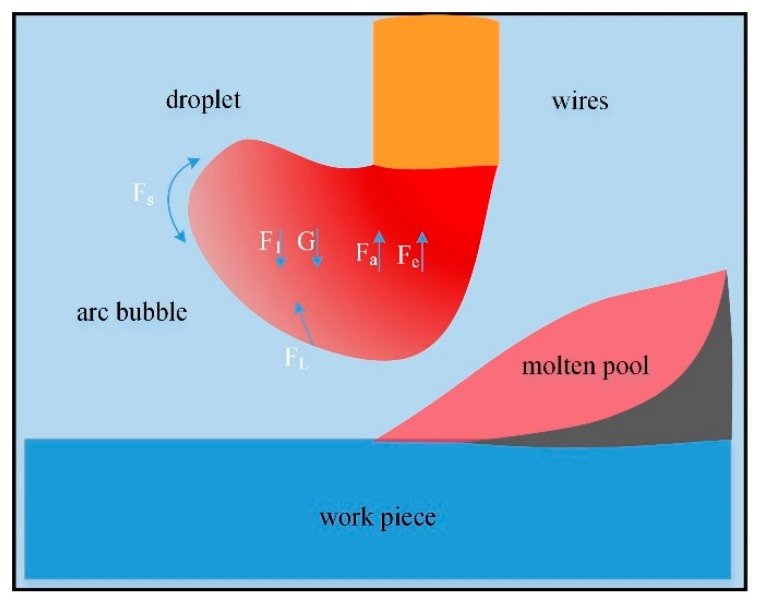
Schematic of forces analysis for globular repelled step.

Secondly, in both droplet transfer modes, repulsion step was the first step. The stress state of the droplet before the transition had great influence on the droplet transfer mode. The schematic of forces analysis for globular repelled step was shown in Figure 14. According to Guo et al. [17], a droplet was mainly subjected to six forces during welding in water based on the metal transfer static force balance theory, which were electromagnetic force (F_e_), surface tension (F_s_), gravity (G), plasma drag force (F_1_), the vaporization force (F_a_) and the gas flow drag force (F_L_). When welding in water, the bubbles were small and the drag force (F_L_) acted transitorily. The droplet transferred into the molten pool under static-force balance theory (SFBT) [18]. The plasma drag force (F_1_) and gravity (G) were detaching force and the others were holding force.

F_L_ existed only in underwater wet welding. It came from the pushing effect of arc bubbles on droplet when it periodically floated up. Chen et al. pointed out that the formula of flow drag force acting on droplets [12]:

(5) $$F_{L}=\frac{1}{2}C_{d}\rho_{g}Sv^{2}$$

where *C_d_* is the flow drag coefficient, *ρ_g_* is the density of the gas inside the bubble, *S* is the contact area between bubble and droplet, ν is the expansion rate or the rising velocity of the bubble.

A larger droplet has a larger contact area(S) with arc bubble. Therefore, it was subjected to a larger F_L_ which caused it to leave the wire at a larger angle, resulted in higher percentage of globular repelled transfer mode. The droplet growth time could reflect its size. The longer the droplet grew, the larger the volume, the larger the area of contact with the bubble (S) and the larger F_L_ it subjected. According to Figure 9, when the filling rate was 15%, the quantity of droplet with larger volume was the most. Continuing to increase the filling rate, the droplet’s volume decreased and then substantially remained almost constant. The ratio of globular transfer mode would first reduce and then remained. However, it is different from Figure 8, where the ratio of globular transfer mode continued to decrease. It could be believed that droplet transition form was not only related to droplet stress, but also related to the shape of molten pool.

**Figure 15:**
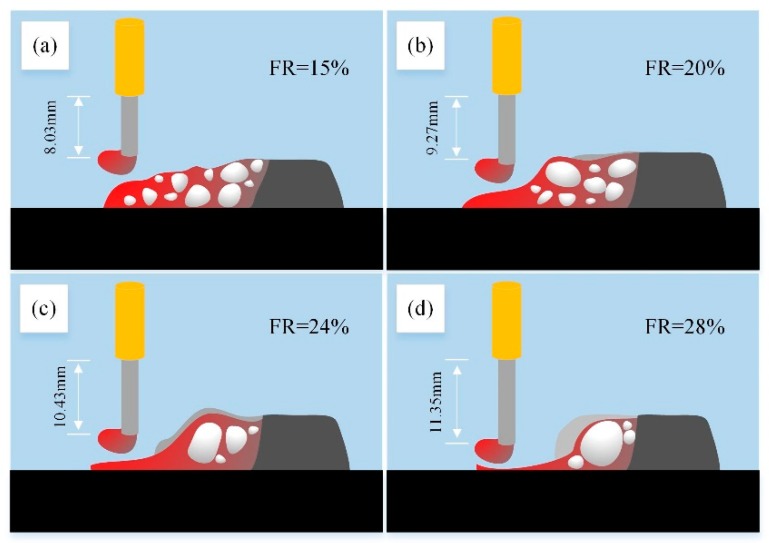
Schematic diagram of molten pool at different filling rate.

The flow behavior of molten pool has direct influence on the final weld forming. However, there were few researches on the behavior of UWW pool. In this study, the molten pool was observed using the X-ray in-situ imaging system and the schematic morphology of molten pool were displayed in Figure 15 (the moment before the droplet transition). The molten pool surface was extremely close to the tip of wires. Violent fluctuation was caused by the bubbles burst and the ever-changing arc force. As the filling rate of wires adding, the shape of welded metal drastically changed. As shown in Figure 15a, the molten pool was full of bubbles dispersedly with a small diameter about 2–3 mm, which increased the volume of the pool. The front of the lack-of-slag molten pool sank down a little bit under the pressure of arc force and liquid vaporization thrust. From Figure 15b,c, the bubbles in the pool gradually diminished since the dehydrogenation of powder and were much more concentrated in the back half as the filling rate increased. The molten pool no longer fluctuated violently under the stronger protection of molten slag. The first half directly below the welding wire gradually became lower and the elongation increased from 9.27mm to 11.35mm. When the filling rate was higher than 15%, the probability of the droplet contacting the pool increased although the droplet size was similar. Therefore, the proportion of surface tension transition increased.

As the filling rate gradually increased, the liquid metal in the molten pool gradually moved back and the part directly below the welding wire gradually decreased because the viscosity of the molten pool decreased. It can be seen from Table 1 that the main components in the molten pool were Ni and a small amount of CaF_2_, Li, Al, Mn, Fe, etc. These components would vaporize in large quantities under the high temperature of the molten pool and the welding arc, which would take away lots of heat and cause the molten pool to cool down. When the filling rate was 15%, due to the lack of slag protection, the elements’ gasification in the molten pool was serious, taking away a mass of heat. Moreover, more slag separated the molten metal from the water environment, greatly delaying the heat conduction of the molten pool. The following exothermic reactions would occur with the Al element in the wire:

2Al + Fe_2_O_3_ → Al_2_O_3_ + 2Fe (6)

2Al + Fe_3_O_4_ → FeAl_2_O_4_ + 2Fe (7)

2Al + 4FeO → FeAl_2_O_4_ + 3Fe (8)

With the increase of filling rate, the increase of Al element would also increase the heat of the molten pool. However, the weld beam was a kind of Ni-based alloy. The viscosity of the Ni-based liquid alloy was negatively correlated with temperature [19,20]. With the filling rate increased, the temperature of the molten pool increased and the viscosity decreased. The liquid metal in the pool was pushed back by the arc force of the welding and the gas gasification thrust. As a result, the height of the first half of the molten pool dropped. At same time, the dry elongation of the wire was increasing. As shown in Figure 15, the shape of first half of molten pool under the welding wire changed from convex to flat or even concave.

**Figure 16:**
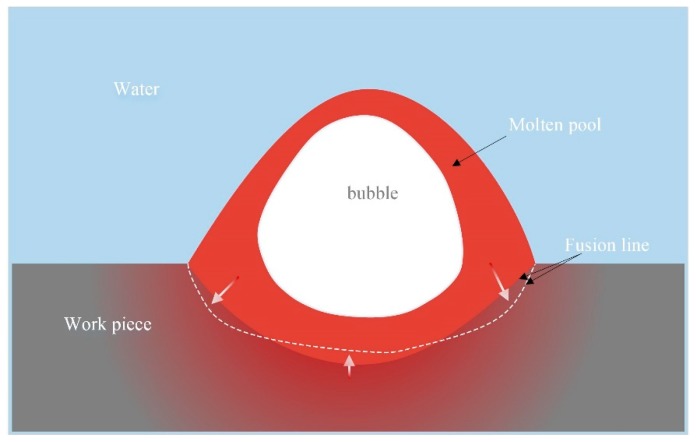
Schematic diagram of fusion line shape change.

Finally, based on the particularity of FCAW, a conjecture on the effect of filling rate on fusion line shape were proposed in this paper: As can be seen in Figure 15, with the increase of filling rate, the molten pool moved back and the size of the inner bubble gradually increased. The larger bubbles in the back half continued to exist during the formation and rupture processes. Under the effect of bubbles, there were more liquid metal at the edge of the bubble and less liquid metal in the central which contacted with the base metal beneath the molten pool. With the scouring of more high temperature liquid metal, the amount of melting base metal beneath the edge of molten pool increased while the counterpart beneath the center of molten pool reduced as shown in Figure 16. Then, the radius of the fusion line decreased. With the increase of filling rate, this phenomenon was much more obvious. However, it is worth mentioning that the weld penetration was mainly determined by the welding parameters. The filling rate had little effect on weld penetration but had significant effect on the shape of fusion line.

## 4. Conclusions

Based on the presented results it was concluded that:

1. with the increase of the filling rate of flux-cored wire, the arc stability first decreased and reached the minimum value when the filling rate was 24%. Then the welding arc became more stable rapidly as the filling rate growing. It was due to the effect of CaF_2_ and other components in the powder on the number of charged particles in the arc.
2. Two droplet transfer modes, globular repelled transfer mode and surface tension transfer mode, were observed in this study. With the filling rate increased from 15% to 28%, the proportion of surface tension transfer mode harshly increased from 51.43% to 83.87%. The increase of molten pool temperature and slag coverage reduced the viscosity of molten pool and affected the shape of molten pool. The wiggle space of the droplet on the tip of the wire was reduced. Resulted in surface tension transfer mode increased.
3. Slag on the weld surface increased and the welds were much flatter and smoother as the filling rate increased. The ratio of depth to width decreased gradually while the reinforcement increased. The fusion line became more regular and the radius of curvature increased under the action of bubbles in the molten pool.

## Figures and Tables

**Table 1:** Flux composition used in experiments (wt.%).

CaF_2_	Al	Mn	LiF
60	20	10	10

**Table 2:** Welding parameters during underwater wet welding

Welding Voltage	Welding Speed	Wire Extension	Wire Feeding Speed	Welding Current
20 V	2.5 mm/s	15 mm	2 m/min	200 A
